# Supplementary figures and images for: Arbuscular mycorrhizal fungi enhance drought resistance in Bombax ceiba by regulating SOD family genes
Source: PeerJ. 2024 Aug 7;12:e17849. doi: 10.7717/peerj.17849 (PMC11316461; doi:10.7717/peerj.17849)

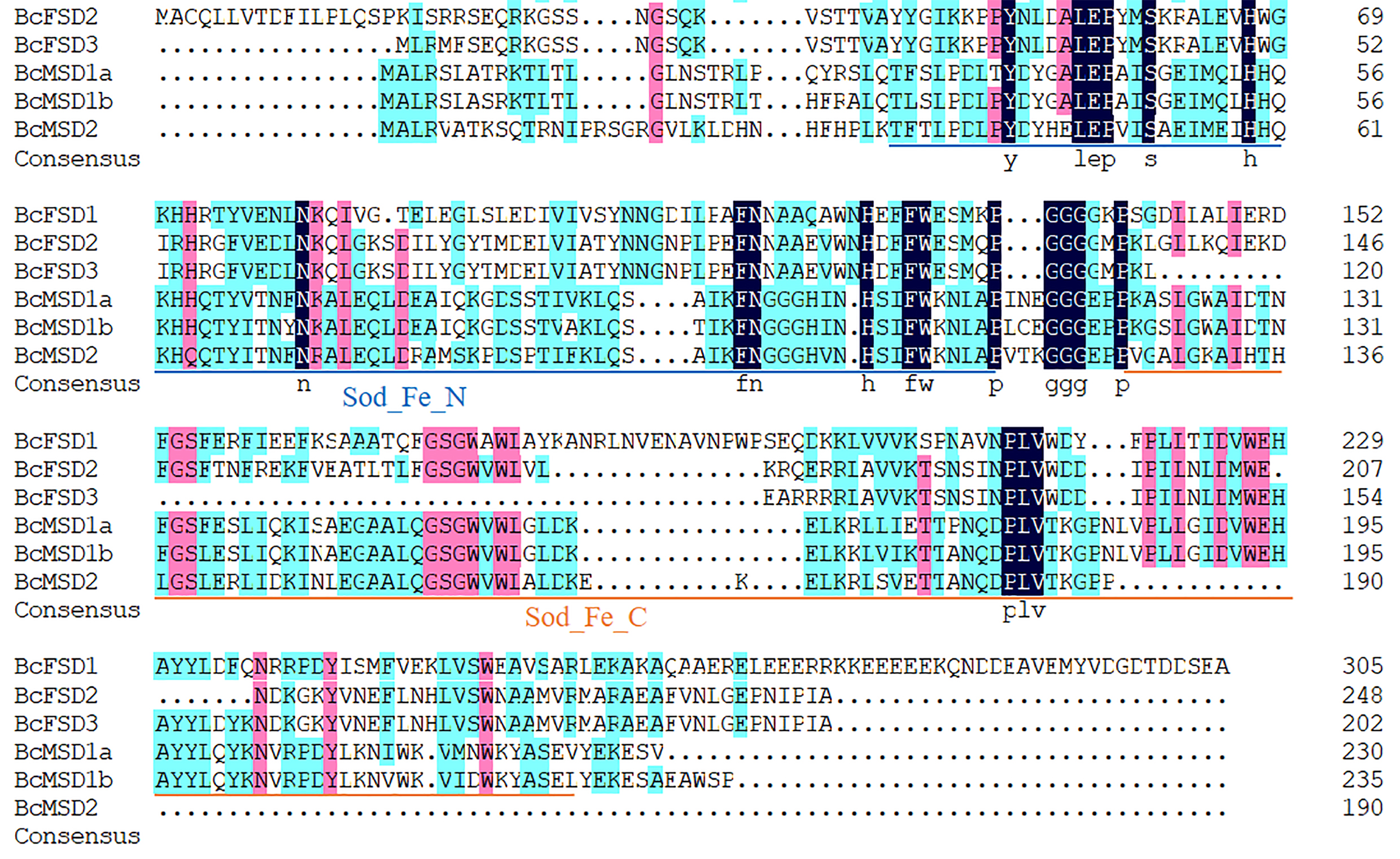

Supplement: Supplemental Information 3 — Figure S1: (a) Multi-alignment of the Fe/Mn-SOD protein sequences; (b) Multi-alignment of the Zn/Cu-SOD protein sequences. Conserved domains in alignment are highlighted with colored lines, and their names are given above the conserved regions; Figure S2: Heatmap and cluster analysis of 14 BcSOD genes relative expression in seedlings that received the AMF and drought stress treatments. [file peerj-12-17849-s003.zip › Supplementary figures and tables/Supplementary Figure 1a.jpg]

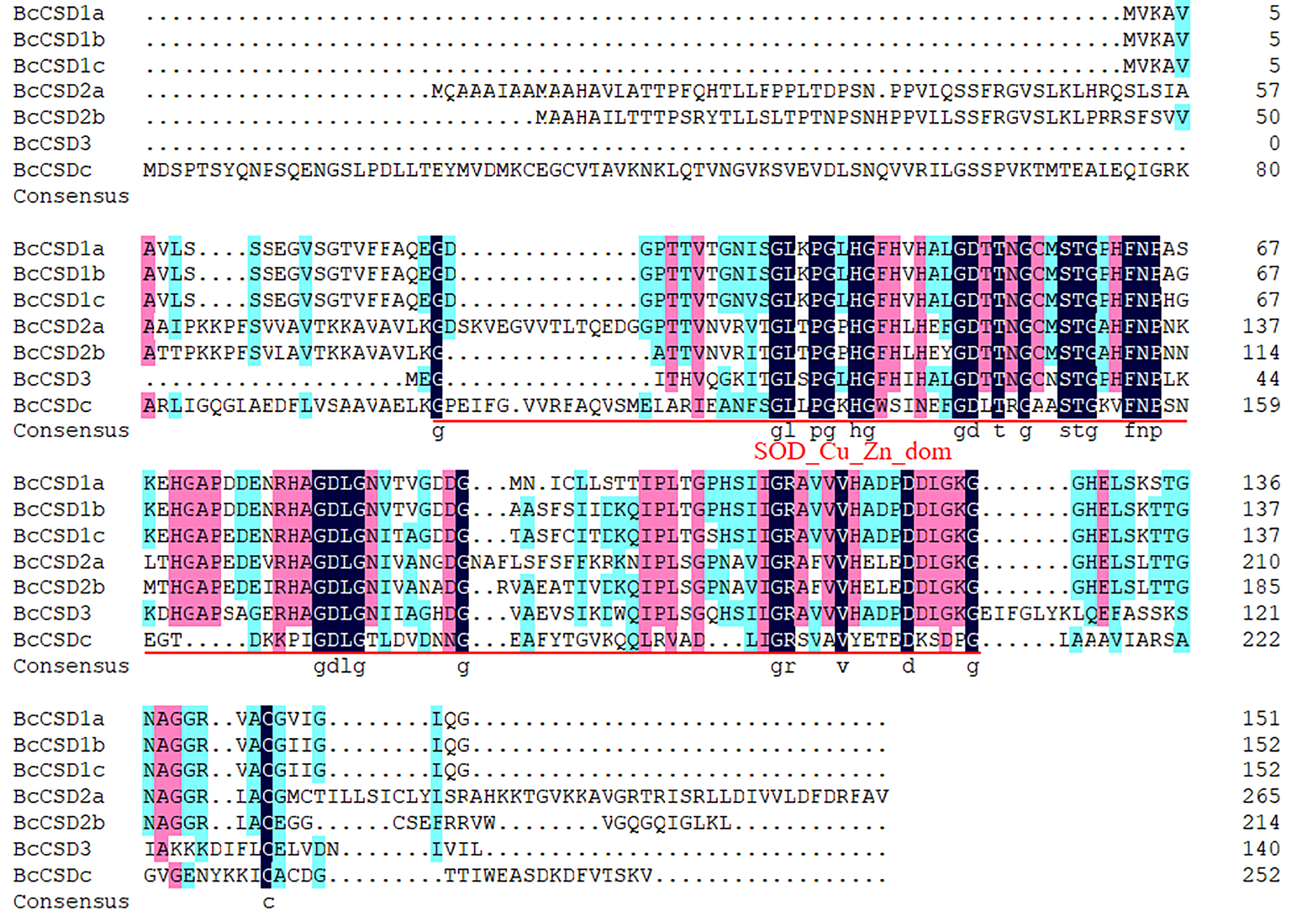

Supplement: Supplemental Information 3 — Figure S1: (a) Multi-alignment of the Fe/Mn-SOD protein sequences; (b) Multi-alignment of the Zn/Cu-SOD protein sequences. Conserved domains in alignment are highlighted with colored lines, and their names are given above the conserved regions; Figure S2: Heatmap and cluster analysis of 14 BcSOD genes relative expression in seedlings that received the AMF and drought stress treatments. [file peerj-12-17849-s003.zip › Supplementary figures and tables/Supplementary Figure 1b.jpg]

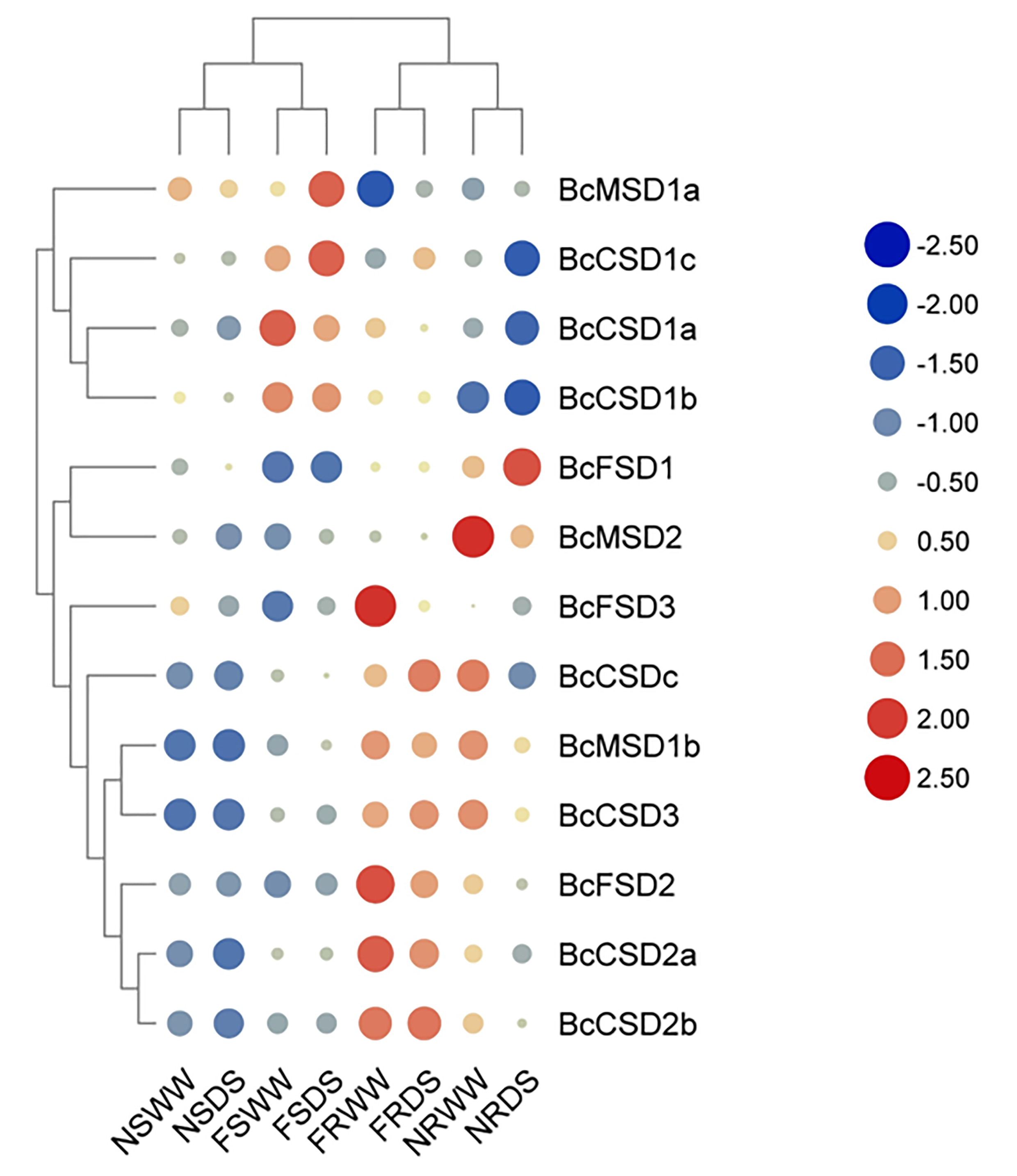

Supplement: Supplemental Information 3 — Figure S1: (a) Multi-alignment of the Fe/Mn-SOD protein sequences; (b) Multi-alignment of the Zn/Cu-SOD protein sequences. Conserved domains in alignment are highlighted with colored lines, and their names are given above the conserved regions; Figure S2: Heatmap and cluster analysis of 14 BcSOD genes relative expression in seedlings that received the AMF and drought stress treatments. [file peerj-12-17849-s003.zip › Supplementary figures and tables/Supplementary Figure 2.jpg]
